# Repurposing Immunomodulatory Drugs to Combat Tuberculosis

**DOI:** 10.3389/fimmu.2021.645485

**Published:** 2021-04-13

**Authors:** Samreen Fatima, Ashima Bhaskar, Ved Prakash Dwivedi

**Affiliations:** ^1^ Immunobiology Group, International Centre for Genetic Engineering and Biotechnology, New Delhi, India; ^2^ Signal Transduction Laboratory-1, National Institute of Immunology, New Delhi, India

**Keywords:** *Mycobacterium tuberculosis*, directly observed therapy short course, repurposed approved drugs, immunomodulators, T cells (Th1/Th2), vaccine, immunotherapy

## Abstract

Tuberculosis (TB) is an infectious disease caused by an obligate intracellular pathogen, *Mycobacterium tuberculosis (M.tb)* and is responsible for the maximum number of deaths due to a single infectious agent. Current therapy for TB, Directly Observed Treatment Short-course (DOTS) comprises multiple antibiotics administered in combination for 6 months, which eliminates the bacteria and prevents the emergence of drug-resistance in patients if followed as prescribed. However, due to various limitations viz., severe toxicity, low efficacy and long duration; patients struggle to comply with the prescribed therapy, which leads to the development of drug resistance (DR). The emergence of resistance to various front-line anti-TB drugs urgently require the introduction of new TB drugs, to cure DR patients and to shorten the treatment course for both drug-susceptible and resistant populations of bacteria. However, the development of a novel drug regimen involving 2-3 new and effective drugs will require approximately 20-30 years and huge expenditure, as seen during the discovery of bedaquiline and delamanid. These limitations make the field of drug-repurposing indispensable and repurposing of pre-existing drugs licensed for other diseases has tremendous scope in anti-DR-TB therapy. These repurposed drugs target multiple pathways, thus reducing the risk of development of drug resistance. In this review, we have discussed some of the repurposed drugs that have shown very promising results against TB. The list includes sulfonamides, sulfanilamide, sulfadiazine, clofazimine, linezolid, amoxicillin/clavulanic acid, carbapenems, metformin, verapamil, fluoroquinolones, statins and NSAIDs and their mechanism of action with special emphasis on their immunomodulatory effects on the host to attain both host-directed and pathogen-targeted therapy. We have also focused on the studies involving the synergistic effect of these drugs with existing TB drugs in order to translate their potential as adjunct therapies against TB.

## Introduction

*Mycobacterium tuberculosis* (*M.tb*) is a deadly pathogen, which infects a large cluster of the population globally and is the cause of the maximum number of deaths due to a single infectious agent (1). The World Health Organization (WHO) has reported that around 10 million people across the globe suffer from active TB infection; with the mortality rate of around 1.3 million (2). Nearly one-fifth of the mortality due to TB is because of the emergence of drug-resistant strains, which do not respond to the frontline anti-TB drugs. The rise in drug-resistance is an alarming situation and has made the control of TB even more challenging. Current treatment of TB involves administration of multiple antibiotics for a minimum period of 6 months for drug-susceptible TB and requires more than two years of treatment in case of drug-resistant TB. This treatment regime is lengthy and is associated with severe side effects such as dampening of the immune system, organ toxicity and emergence of drug-resistance (3).

To mitigate the challenges of dealing with TB, we are in urgent need of novel drugs with an action mechanism that can treat as well as shorten the treatment regime for both drug-susceptible and drug-resistant strains, which may be better tolerated and may increase adherence to the therapy. Recently, three new drugs, bedaquiline, delamanid and pretomanid have been approved by the Food and Drug Administration (FDA) against TB (4, 5). However, it is very difficult and tedious to develop new efficacious TB treatment regime owing to the time taken and the cost incurred in various processes involved. Therefore, the pharmaceutical industries as well as the researchers are focusing on identifying novel drug and target interactions using pre-existing drugs which have been used in the treatment of different diseases, a practice called drug repurposing.

**Figure 1:**
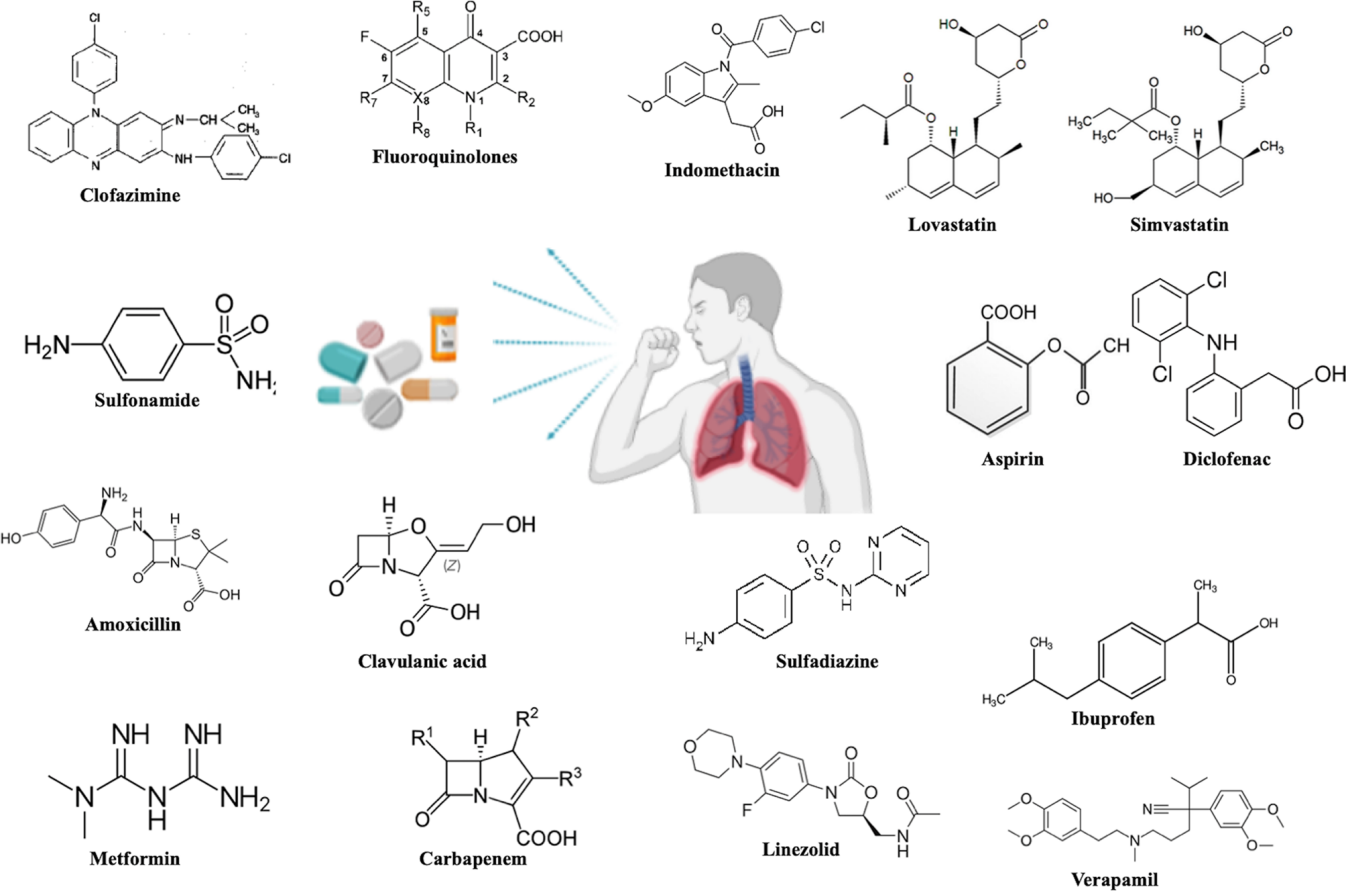
Chemical structures of the proposed repurposed drugs under investigation against TB disease.

**Figure 2:**
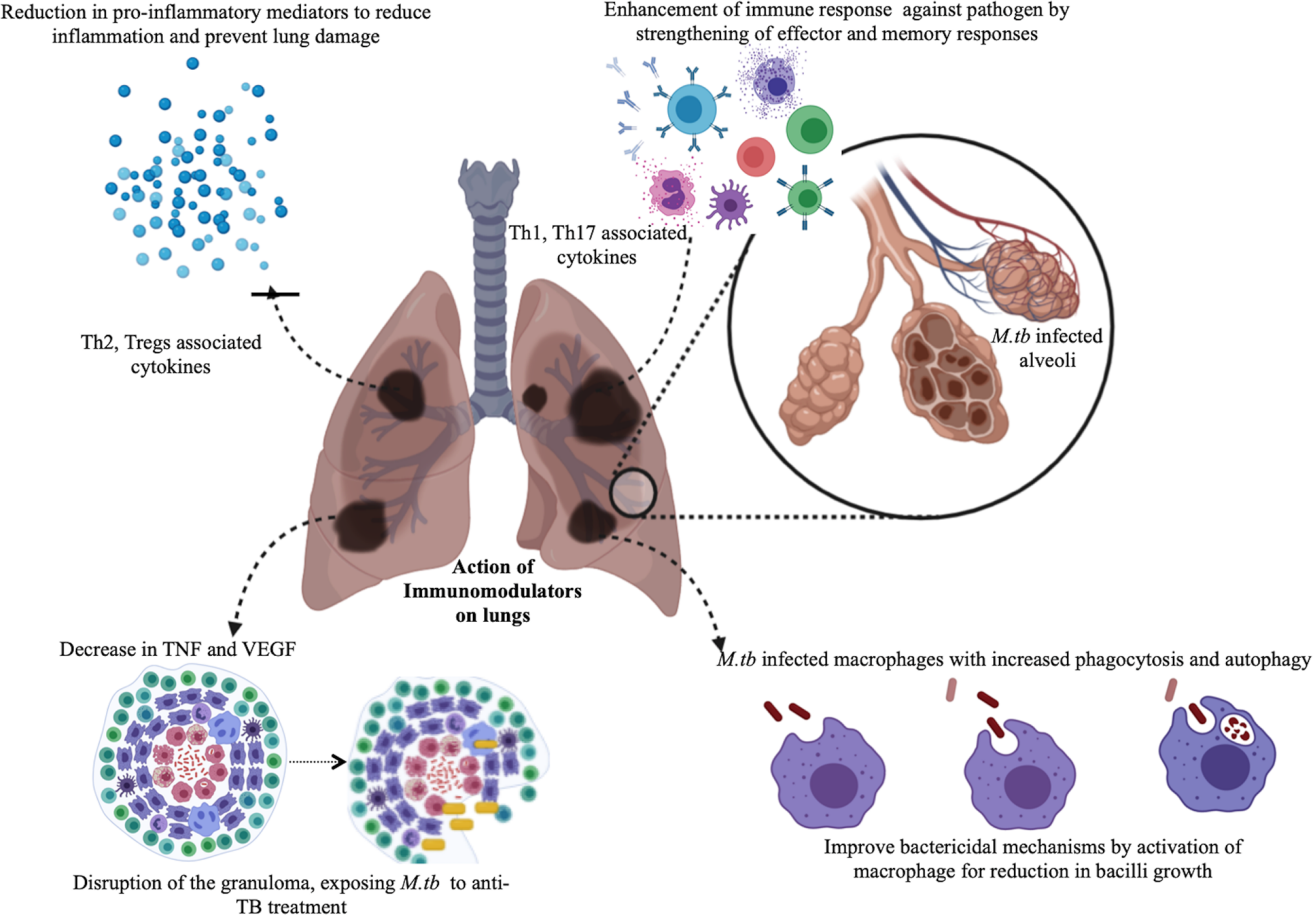
Mechanism of action of immuno-modulatory drugs.

Drug repurposing, also known as drug repositioning involves identifying new therapeutic usages of already established and approved drugs. This strategy encompasses a lower risk of failure, reduces the time required for new drug development, involves comparatively less investment and may lead to the discovery of novel targets which may be used for further research in the pharmaceutical field (6). The most successful example of drug repurposing is Sildenafil (7). It was initially developed for use as an anti-hypertensive drug but has gained huge popularity as a medication against erectile dysfunction. Its repurposed use as an inhibitor of phosphodiesterase 5 is a consequence of a serendipity (7). Later, drugs such as thalidomide and its derivatives like lenalidomide were successfully used as repurposed drugs for the treatment of diseases such as Erythema Nodosum Leprosum (ENL) and multiple myeloma (8). The success of these drugs by serendipity has led to the ongoing serious efforts by researchers in discovering new roles of pre-existing drugs in different disease contexts. In TB, drug repurposing offers a very attractive strategy to deal with the emerging challenge of drug resistance and to discover drug combinations that may shorten the duration of treatment ultimately preventing the development of resistance and promoting adherence to the treatment. Drug repurposing is by far transforming translational research by assuring total safety and efficacy while cutting short the time invested in passing the regulatory hurdles and thus ensuring that the drug reaches the clinic within 3-4 years. Moreover, there has been ongoing research on the use of immuno-modulators as an adjunct therapy with the conventional DOTs (Directly Observed Treatment, Short-course) treatment with the sole purpose of reducing the duration of treatment as well as pulmonary toxicity (9). This adjunct therapeutics has been designed so as to prevent reinfection and also reactivation of the TB disease. Many of the repurposed drugs are an attractive target to be used as immuno-modulators as there is no safety concern involved with them. Their use as immune modulators along with the standard anti-TB regimen may achieve total elimination of the pathogen in a short time. The chemical structure of the various drugs repurposed for TB has been shown in **Figure 1**. Immunomodulators are natural or synthesized compounds that activate or suppress the immune system by the release of either pro-inflammatory or anti-inflammatory cytokines in order to help the immune system deal with a pathogen more effectively. Pro-inflammatory responses by cytokines released by T cells such as IFN-γ, TNF-α in association with IL-6, IL- 1 and chemokines such as CCL5, CCL9, CXCL10, and CCL2 attracts immune cells at the site of infection and lead to the effective elimination of the pathogen (10). The pro-inflammatory cytokine response is mainly responsible for initiating a cascade of events that ultimately leads to the killing of *M.tb*. The immunomodulators act on different immune cells such as neutrophils, macrophages, lymphocytes, natural killer (NK) cells to exert their effector responses aimed at clearing the bacteria from the host. The mechanism of action of immuno-modulators has been shown in **Figure 2**.

These immunomodulators have gained tremendous attention in anti-TB therapy as these compounds when administered together with the DOTS regime helps in the early clearance of the infection as well as aids in the prevention of drug-resistance development. Many of the immunomodulators help mask the side effects of the harsh anti-TB antibiotic therapy.

Here, in this review, we discuss drugs, which display promising effects against TB and hence have been repurposed for use against TB. The drugs that we have discussed appear to be the most significantly studied in case of TB. We also highlight their mechanism of action along with any study if present for their use as immuno-modulators as an adjunct therapy against TB (**Table 1**).

**Table 1:** Repurposed drugs with their year of introduction and status in TB treatment.

Name of drug	Year of introduction	Status of the drug	Properties and efficacy against TB
Clofazimine	In 1969 for the treatment of leprosy	Approved	Reduces the treatment length for drug-resistant TB and displays immuno-modulatory properties
Statins	In 1959 for cardiovascular diseases	Phase 2 clinical trials	Anti-inflammatory and immuno-modulatory
NSAIDs	In 1969 for the treatment of rheumatoid arthritis	Phase 3 clinical trials	Anti-inflammatory and immuno-modulatory
Fluoroquinolones	In 1962 for the treatment of bacterial infection	Approved f	By inhibiting the replication and transcription of bacterial DNA
Linezolid	In 1990s for vancomycin- resistant *Enterococcus faecium* infections	Approved	Acts as a protein synthesis inhibitor
Verapamil	In 1968 for treating blood pressure	Phase 2 clinical trial	Calcium efflux blocker, which reduces the duration of TB therapy.
Metformin	In 1922 to treat diabetes	Phase 2b clinical trial	Immunomodulatory
Amoxicillin/clavul anic acid	In 1974 for treatment of bacterial infections	Phase 2 clinical trial	Prevents bacterial cell wall synthesis
Carbapenems	In 1976 to inhibit beta lactamase enzyme	Phase 2 clinical trials	Target the cell wall of *M.tb* bacteria
Sulphonamides and their derivatives	In 1956 against gram positive and gram negative bacteria	Approved	Used as combination therapy against drug resistant TB

## Repurposed Drugs for Anti-TB Treatment and Their Immunomodulatory Properties

**Figure 3:**
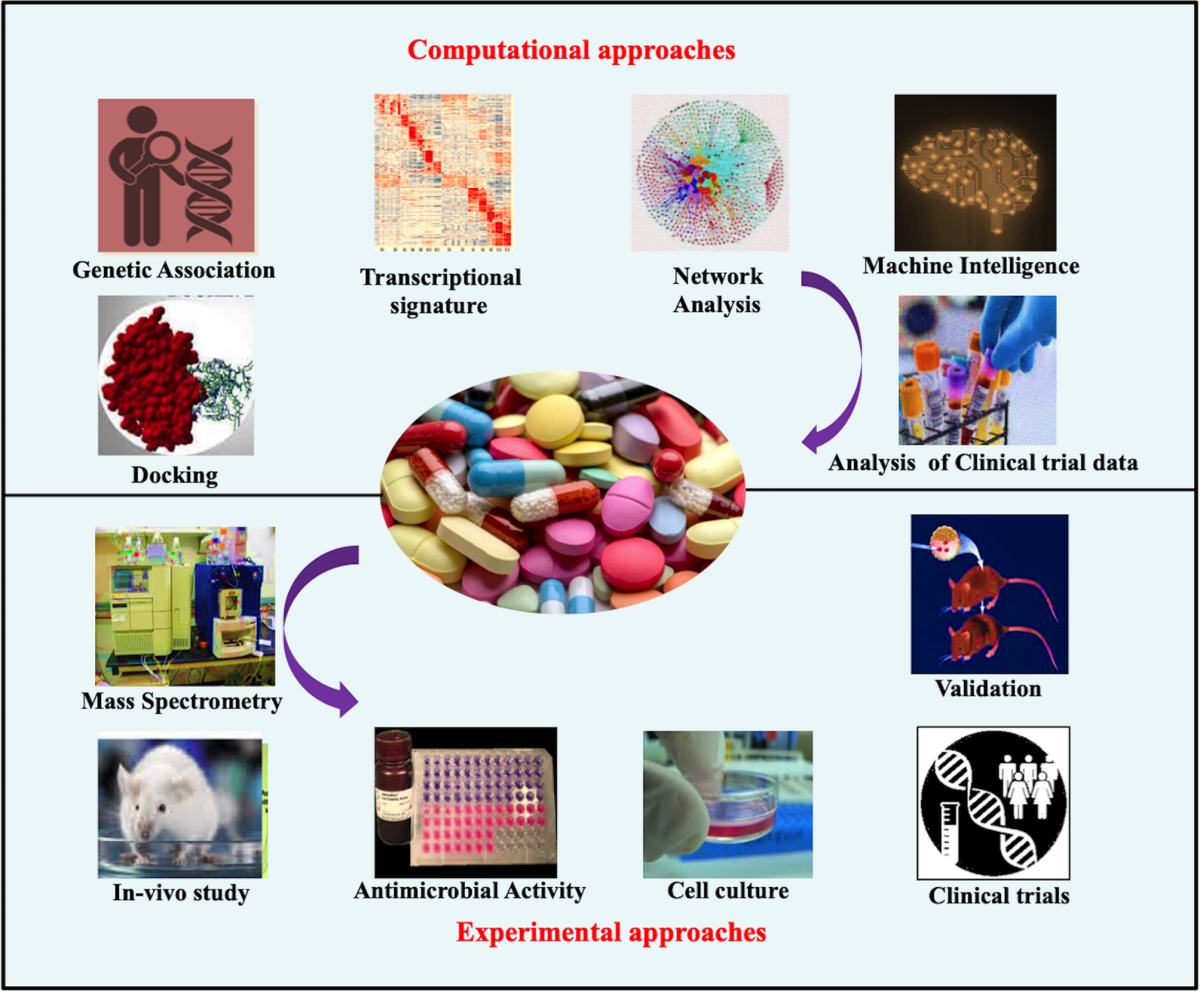
Different computational and experimental approaches used in drug repurposing.

With very slow development in the addition of novel drugs against TB and the fast emergence of drug resistance among TB patients, there is a need to focus on repurposing drugs for better treatment outcome against TB. WHO has recommended the inclusion of repurposed drugs such as fluoroquinolones, linezolid, clofazimine, and carbapenems, among many others, for the treatment of drug-resistant TB. Here, we discuss each of these repurposed drugs along with their mechanism of action and any immunomodulatory role if known to date. Different approaches used to repurpose the drugs against different diseases have been summarized in **Figure 3**.

### Clofazimine

Clofazimine, a riminophenazine antibiotic was discovered in Dublin in the 1950s and was originally introduced in 1969 for the treatment of leprosy (11). Recently, it has been repurposed for the treatment of drug-resistant TB after a study which stated that including clofazimine in anti-TB regime could treat MDR-TB in 9-12 months and has been recommended by WHO as a second-line drug along with other first line treatments (11). Clofazimine works like a pro-drug, which releases reactive oxygen species (ROS) upon re-oxidation by oxygen after initially being reduced by NADH dehydrogenase (NDH-2) (12). Clofazimine apparently competes with menaquinone (MK-4) for its reduction by NDH-2 (13). Clofazimine also exerts its anti-mycobacterial as well as anti-inflammatory properties by a Ca^2+-^independent increase in mycobacterial PLA2 and by its effects on potassium channels (14, 15). However, it is independent of the C-type phospholipases of *M.tb* (16). Owing to its efficacy and negligible toxicity in the treatment of drug-resistant strains in mice model studies and in clinical trials, it comes out as a promising drug candidate for TB management (17, 18). In different clinical trials in Bangladesh and China, clofazimine has shown to reduce the treatment duration of MDR-TB (17) and is recommended by WHO for treatment of drug-resistant TB along with other drugs. Clofazimine is reported to display immuno-modulatory properties by enhancing T_CM_ (Central Memory T cells) responses while reducing T_EM_ (Effector Memory T cells) population by blocking KV1.3^+^ potassium ion channel on the surface of T_EM_ (18, 19). Depending upon their differentiation state, homing potential, duration of survival and production of three major cytokines-IFN-γ, TNF-α and IL-2 (20). Memory T cells help in providing protective immunity against TB. Clofazimine is inexpensive, compared with other drugs in an MDR-TB drug-regimen and seems to be very promising as drug of future, for TB.

### Statins

Statins are HMG-CoA reductase inhibitors that have recently been explored for their anti-tubercular effects (21). Statins have been prescribed to hyperlipidemic patients in order to reduce the risk of stroke and other cardiovascular diseases. Recent studies have demonstrated that statins have anti-inflammatory and immunomodulatory properties as well (22). Statins prevent TB by blocking the HMG-CoA reductase thereby reducing cholesterol synthesis and accumulation. Also, statin inhibits the process of phagocytosis, which is essential for the uptake of *M.tb* inside macrophages (23). The pioneering study to establish the effect of statin on TB infection was conducted 20 years ago where it was observed that statins, in particular, fluvastatin plays an immunomodulatory role by modulating the Th1 and Th2 cytokine responses, inducing the release of pro-inflammatory cytokines, IL-1β, IL-18 and IFN-γ, and also leads to as the activation of autophagy and apoptosis (24).

Recently, Parihar et al. reported that PBMCs and monocyte-derived macrophages (MDMs) isolated from familial hypercholesterolemia patients undergoing statin therapy (for a minimum period of six months) were more resistant to *M.tb* infections then those from patients not on statin treatment (25). It was also reported that statins treatment reduced TB pathogenesis and disease severity in mice. Simvastatin treated bone marrow-derived macrophages (BMDMs) displayed significantly reduced bacterial burden compared to the untreated cells owing to increased phagocytosis and autophagy. In the mice model, treatment with statins showed a 10-fold reduction in the bacterial load in the major organs infected (spleen, liver, and lungs) as compared to the untreated mice (25). Later in the same year, it was reported that atorvastatin and simvastatin reduced mycobacterial burden up to 75 percent and showed a synergistic effect with front-line anti-TB drug rifampicin (RIF) in the murine model of TB (26, 27).

Dutta et al., in 2016 studied the effect of simvastatin with front-line anti-TB drugs as an adjuvant for its role in reducing the duration of treatment in mice model of TB (28). Simvastatin was observed to significantly increase the anti-mycobacterial activity of first-line antibiotics while significantly reducing the time required to achieve sterile clearance in the lungs hinting at its use as an adjuvant with anti-TB treatment.

Apart from the anti-mycobacterial activity, studies have also shown the immunomodulatory activity of this drug against TB (29). Simvastatin has been shown to increase the number of natural killer (NK) T cells, induce the secretion of pro-inflammatory cytokines and enhance the expression of co-stimulatory molecules on monocytes together with an increase in autophagy and apoptosis which ultimately leads to a steady decrease in bacterial load (29). In 2019, Dutta et al. performed another study with HMG-CoA inhibitors such as pravastatin and fluvastatin along with simvastatin with first-line anti-TB drugs to evaluate their potential as adjunct agents. They concluded that of all the statins tested; pravastatin was the most potential to be used as an adjunct with the least toxicity (30). They also concluded that the addition of statins to first-line drugs reduce the duration of the therapy thereby proposing this therapy for the treatment of TB in human patients.

Recently it has been reported that during TB treatment, greater than 99% of the bacteria clear up within 3 weeks of treatment. However, less than 1% of bacteria become non-responsive to conventional antibiotics and remains in a metabolically inactive state. These non-responsive bacteria hibernate in Mesenchymal Stem Cells (MSCs), and are refractive to conventional antibiotics. Upon infection in MSCs, the *M.tb* population migrates to the cytosol where they induce lipid synthesis. Finally, *M.tb* slides into lipid droplets where they hibernate and use the host’s lipids as a carbon source. A macrophage is a natural host for active TB, whereas MSCs are the host for dormant TB. *M.tb* in MSCs can be killed by inducing autophagy or by inhibition of lipid synthesis (31). Therefore, it has been shown that the addition of rapamycin or statin along with conventional antibiotics dramatically reduce the length of TB treatment eliminating both replicating and hibernating dormant bacteria, in turn reducing the possibility of generating drug resistance. The statin, pravastatin, is in Phase 2b clinical trials. Despite, more than two decades of research on the use of statins as an anti-TB agent, the initial results of clinical studies are very uncertain. However, considering the promising results in mice models, further clinical trials to investigate the effect of Statins in the treatment of TB are recommended.

### NSAIDs

Non-Steroidal Anti-inflammatory Drugs (NSAIDs) are a class of drugs that are used to treat inflammation, pain and fever (32). They reduce inflammation by inhibiting the synthesis of prostaglandins, which mediate the inflammatory process. NSAIDs target the Cyclooxygenase enzymes, COX1 and COX2, which synthesize the prostaglandins from arachidonic acid. NSAIDs were initially used as analgesic, antipyretic and anti-inflammatory drugs. However, their effect has recently been explored in cancer and neurodegenerative diseases (33). The main NSAIDs used in TB treatment in mice model are diclofenac, Ibuprofen and Aspirin, and Indomethacin. The main mechanism, through which NSAIDs work during TB treatment, is by reducing the inflammation caused by the influx of monocytes, lymphocytes and neutrophils (34). As these cells produce a high amount of prostaglandins (PGE2), which causes inflammatory effects, NSAIDs attenuate the disproportionate inflammatory response caused by migration of these cells during active TB and thus may help in the improvement in the disease outcome (35).

Diclofenac, mostly used to treat arthritis and gout, has recently been used as an antimicrobial drug. A study by Dutta et al. showed that Diclofenac treated mice displayed reduced bacterial burden and disease pathogenesis as compared to the control group (36). Diclofenac also shows a synergistic effect with Streptomycin in mice model of TB (37). Diclofenac has been known to dampen the host immune system by inhibition of Kv1.3 expression in activated macrophages and T lymphocytes. Diclofenac treatment in macrophages leads to decreased iNOS levels thereby hindering their activation (38). However, no immune study involving diclofenac has been conducted in respect to TB.

Indomethacin is COX-inhibitor, which does not differentiate between COX-1 and COX-2. Recently its use as an immunomodulator to balance the T cell phenotype during TB has come to the fore-front because of its immunosuppressive nature. Since, TB is a disease characterized by both infection and inflammation; anti-inflammatory drugs such as NSAIDs improve the disease outcome in severely ill patients. Hernandez-Pando et al. reported the use of indomethacin in regulating T cell imbalance in the granuloma during the course of the disease (39). In another study, in mice immunized with *M. vaccae*, pre-treatment with indomethacin induced better response than in non-treated animals (40).

Ibuprofen, like indomethacin, is an indiscriminating COX-inhibitor. Ibuprofen has been reported to promote survival of *M.tb* infected mice while decreasing the number and size of lung lesions because of the low bacterial burden (41). Moreover, there was reduced infiltration of neutrophils in ibuprofen treated mice as compared to the control group. As reported by Vilaplana et al., combine therapy with Ibuprofen and isoniazid reduced the neutrophilic invasion but aspirin showed the opposite effect. Therefore, they suggested the use of ibuprofen and recommended not using aspirin during TB infection (41). Another group (Byrne et al.) also confirmed the same results (42). However, Byrne et al. in yet another follow-up study confirmed that both ibuprofen and aspirin can be used along with the first-line anti-TB drugs to shorten the treatment course (43). In a study in TB patients who had already been treated by first-line anti-TB drugs, aspirin significantly lowered the serum uric acid concentrations to almost normal levels during the treatment of arthralgia (44). A few years later, Horsfall et al. showed that while treating the arthralgia patients with pyrazinamide, along with anti-arthralgia drugs (aspirin or allopurinol), the aspirin-treated group showed better disease outcome (45). The potential role of aspirin has been investigated in another randomized human trial, in the early treatment of TB meningitis along with the immune-suppressant, dexamethasone. This study established the adjunct potential of aspirin as host-directed therapy that inhibits thromboxane-A2 to reduce new brain infarcts (46). To conclude, all studies on NSAIDs establish their potential as immuno-modulators that can be favorable if given as co-therapy during TB treatment. Their protective potential is facilitated by their anti-inflammatory properties. They work by improving the effectiveness of antibiotics and have some bactericidal potential as well.

### Fluoroquinolones

Fluoroquinolones (FQs) are antibiotics, which kill the bacteria by binding to and inhibiting the function of topoisomerase II and IV enzymes. FQs penetrate into the lipid bilayer of the bacteria to exert their functions (47). WHO has recommended the use of FQs (moxifloxacin, gatifloxacin, levofloxacin), for treating MDR-TB, as second-line anti-TB drugs (48). Moreover, FQs also mediate change in the host immune responses (49). Riesbeck et al. discovered that FQs induce the secretion of IL-2 (50) in mice through the activation of transcription factor NFAT-1 (51). Some FQs, (ciprofloxacin, moxifloxacin, levofloxacin, trovafloxacin, and grepafloxacin) induce the expression of IL-2 in monocytes stimulated by LPS while preventing the expression of TNF-α (52). FQs also suppress the production of pro-inflammatory cytokines. Katsuno et al. reported that in the presence of IL-18, FQs reduce the secretion of IFN-γ (53). Recently, it has been reported that by reducing the expression of CD40, norfloxacin lowers the production of IFN-γ in Langerhans cells (54).

Similar findings have been reported in different diseases such as cancer and viral infections in the mice model (55, 56). Healthy individuals consuming ciprofloxacin and moxifloxacin display decreased IFN expression in the lymphocytes (57, 58). IL-12 is an essential cytokine for Th1 cell responses (59). It has been documented that in patients with TB, levofloxacin and inhibits IL-12 production (58).

Matsui et al. have reported that treatment with norfloxacin leads to the reduction in Th2 responses by limiting IL-4 production (54). FQs such as moxifloxacin and ciprofloxacin reduce IL-4 expression in PBMCs from healthy individuals (57). On the contrary, in diseases, FQ treatment leads to the increased production of IL-4 and IL-10 (60–65). Although FQs are known to exert anti-inflammatory functions, limited work has been done to explore their immunomodulatory properties in TB.

### Linezolid

Linezolid is a synthetic antibiotic, which are used to treat several gram-positive bacterial infections. They inhibit bacterial protein synthesis by preventing the formation of the translation initiation complex (66). Linezolid is the first member of the oxazolidinone class of antibiotics, which were initially used against plant infections. A few years later, their antibacterial properties were documented (67). Due to the ineffectiveness of DOTs therapy in treating drug-resistant bacteria, WHO has recommended the use of linezolid as a potential repurposed drug to treat patients infected with MDR-TB or XDR-TB (68) after it being accepted by the US Food and Drug Administration (FDA or USFDA) for antibacterial use in 2000. Several studies demonstrate the *in-vitro* and *in-vivo* effect of linezolid in the treatment of MDR-TB both in humans and mice which proved its effectiveness in treating DR-TB (69–77); though some studies also report that it exhibits various side effects such as neurotoxicity and blood toxicity (78). Nevertheless, linezolid administration has better adherence, better efficacy and is well tolerated by the DR patients (79). A recent report states that combination therapy of bedaquiline with linezolid is safe for treatment of pregnant DR patients with no reported toxicity in the fetus (80). Therefore, in spite of the drawbacks such as neurotoxicity and blood toxicity, linezolid could be used to treat drug resistance in patients where survival is a priority. However, the dose and duration of treatment need more optimization.

Several *in-vitro* and *in-vivo* studies have established the immunomodulatory nature of linezolid. It has anti-inflammatory effects as it suppress the phagocytic ability of macrophages (THP-1) after infection with heat-killed *E. coli* (81). In the mice model of diseases, such as pneumonia and sepsis, the immunomodulatory effects of linezolid have been extensively studied and almost all studies report that linezolid reduces the damage caused due to excessive inflammation by long term production of pro-inflammatory cytokines. Moreover, it reduces the production of cytokines such as interleukin-1β (IL-1β), IL-6, IL-8, IFN-γ, and TNF-α and reduces the infiltration of neutrophils and monocytes at the infection site as demonstrated by various mice and human studies (82–87).

In a study conducted on 52 patients infected with Methicillin-resistant *Staphylococcus aureus* (MRSA), it was reported that the majority of the patients showed a significant decrease in fever in 3 days, despite being culture positive when treated with linezolid, as compared to the untreated cases, which take a week for the reduction in fever (88). This may be due to the anti-inflammatory properties of linezolid. Another report by Danin et al. studied the effect of linezolid on cytokines production in periapical tissues of teeth (89). They reported that linezolid had a different effect on proinflammatory cytokines. While IL-1ra level was decreased, IL-6, and TGF-β level remained the same. These studies establish that linezolid has significant immunomodulatory properties. However, the effect of linezolid treatment on the host immune system during TB is highly understudied.

### Verapamil

Verapamil is an efflux pump inhibitor (calcium ion channel inhibitor), which is used to treat patients with high blood pressure and cardiac disorders. It inhibits the entry of calcium into the calcium channels present in the heart muscle cells and those in arteries (90). This causes relaxation of heart muscles and vasodilation. It also improves the delivery of oxygen to the heart and thus helps in treating angina patients (91). An initial study by Gupta et al. suggested that verapamil together with standard anti-TB therapy improves the bacterial clearance in *M.tb* infected mice, reduces the time of treatment and decreases the disease relapse rates to a much greater extent than in mice undergoing standard treatment, suggesting an adjunct role for verapamil in anti-TB therapy (92). Gupta et al. also suggested that administration of verapamil together with bedaquiline reduces the bacterial load in *M.tb* infected mice and therefore calcium efflux blockers can be explored as adjuncts in TB therapy (93). After this pioneering work, many follow up studies also confirmed the protective role of verapamil in TB therapy (94–96). These studies demonstrated other mechanisms of protection conferred by verapamil such as by increasing the bioavailability of bedaquiline (94) and by disturbing the mycobacterial membrane energetics (95). It also displayed a protective adjunct effect in combination with front-line anti-TB drugs in rifampicin-resistant strains of *M.tb* (96). However, there is not much literature on the effect of verapamil on the immune system and therefore studies are needed to establish the role of verapamil as an immunomodulator in TB despite it showing promising results in TB treatment.

### Metformin

Metformin is a very old drug, which is used to treat type 2 diabetes. Metformin acts by decreasing the production of glucose in the liver, minimizing the absorption of glucose and increasing its peripheral utilization. Metformin functions by AMP-activated protein kinase (AMPK) dependent and independent mechanisms. The other proposed mechanisms are by inhibiting mitochondrial respiration by blocking NADH: ubiquinone oxidoreductase (Complex I) of the mitochondrial electron transport chain or by targeting the mitochondrial glycerophosphate dehydrogenase (97). In the mitochondria, upon inhibition of Complex I, activation of 5′- adenosine monophosphate-activated protein kinase (AMPK) takes place. AMPK upon activation tries to restore the energy balance of the cell by activating catabolic pathways (energy-generating) for ATP-generation and, stopping the functioning of the anabolic mechanisms (energy-consuming). Metformin increases AMPK activation, which in turn inhibits the mammalian target of rapamycin (mTORC1), which eventually shifts the cellular state to catalytic form and leads to fast utilization of glucose to maintain the energy homeostasis in the cell (97).

Singhal et al. (98) reported the use of metformin as an adjunct therapy against TB. In THP-1 cells and human monocyte-derived macrophages (hMDMs), treatment with metformin reduced mycobacterial growth, which was AMPK dependent as cells deficient of AMPK did not show this effect (98). In the *in-vivo* mice model, metformin treatment increased the efficacy of standard anti-TB drugs and showed reduced disease pathology compared to those treated with isoniazid (INH) and ethionamide alone. Treatment with metformin enhanced the protective immune response and increased ROS production. The drug proved effective in eliminating drug-resistant bacterial strains as well by promoting efficient phagosome-lysosome fusion. In human studies, treatment with metformin improved the disease severity in both the two cohorts tested and provided better elimination of the bacteria. This study indicated that metformin could be used as adjunctive therapy for improving the effectiveness of the standard treatment course of TB.

Another recent study by the same group shed some light on the mechanism of protection conferred by metformin (99). Their work demonstrates that metformin educates CD8^+^ T cells and enhances their anti-mycobacterial capacity as the mice infected after being adoptively transferred with metformin-treated CD8^+^ T cells showed a significant reduction in *M.tb* load in the lungs as compared to the control mice. Also, there was a significant difference in the size of the lung between the two groups. They reported that metformin treatment has a major effect on CD8^+^ T cells, which expand to form memory like CD8^+^CXCR3^+^ T cells in mice, which confer long-term by enhancing BCG elicited CD8^+^ T-cell responses. These host protective CD8^+^CXCR3^+^ T cells helped in achieving better clearance of the bacteria and prevented disease reactivation in a major percentage of the population. These results were also confirmed in the human PBMCs. Therefore this study establishes the role of metformin as an agent which educates the CD8^+^ T cell compartment to undergo metabolic programming to form memory like CXCR3^+^ T cells which have better homing capacity and protective potential.

Other groups have also proposed the use of metformin as an adjunct therapy since, in human cohorts studies, there was a significant reduction in the mortality rate in patients receiving both metformin and DOTs treatment (100–103). Other studies also establish the role of metformin in manipulating the host immune response against TB. For instance, metformin affects the number of total neutrophils and white blood cells with an increase in the ratio of monocytes to lymphocytes in the circulation (104). Treatment with metformin leads to up-regulation of genes for ROS and causes culture conversion through the process of autophagy (98,103,https://www.frontiersin.org/articles/10.3389/fmicb.2020.00435/full#B19).

### Amoxicillin/Clavulanic Acid

Back in the 1940s, it was discovered that penicillin, a β-lactam antibiotic, was non-inhibitory to *M.tb in vitro* (104). Further research revealed that *M.tb* is impervious to β-lactams *in-vitro* due to the presence of *M.tb* penicillinase, which is encoded by BlaC gene (105). Over the years, research has revealed that a β-lactam antibiotic when combined with a β-lactamase inhibitor maintains its potency (106). So, amoxicillin, an antibiotic of the beta-lactam family of antibiotics, in combination with clavulanate, a beta-lactamase inhibitor has been widely prescribed for oral administration as a broad-spectrum antibiotic for treating a variety of bacterial infections (105). Combining amoxicillin with clavulanate has widened the spectrum of usage of amoxicillin against β-lactamase-mediated resistant bacterial strains such as *M.tb* (106). The peptidoglycan cell wall synthesis requires the action of DD-transpeptidases enzymes that are basically penicillin-binding proteins (PBP). Amoxicillin works by binding to these enzymes thereby blocking the peptidoglycan cell wall synthesis, which eventually leads to bacterial death (107). Clavulanic acid has no antimicrobial activity of its own and works by stopping the bacteria from destroying amoxicillin (108).

Subsequently, a number of studies have been carried out all around the globe to understand and examine the *in vitro* efficacy of amoxicillin-clavulanate against clinical *M.tb* isolates. Owing to the absence of established baseline sensitivity breakpoints of amoxicillin-clavulanate against *M.tb*, the ratio of MICs in these studies have been quite varied, varying from 2:1 amoxicillin to clavulanate concentration (109) in one study to almost 13:1 amoxicillin to clavulanate concentration (110) in another. However, it was established that even at the lowest oral dosage of 375mg, amoxicillin-clavulanate were concentrated in bronchial mucosa, most likely producing lung tissue levels enough to inhibit common respiratory pathogens (111). Researchers have also studied correlations between amoxicillin-clavulanate efficacies on various resistance strains of *M.tb*. Amoxicillin/clavulanic acid have been proposed in combination therapy with second-line anti-TB drugs for the treatment of DR-TB owing to its low cost and fewer side effects by WHO and is chosen to be included in group 5 antibiotics (112). Hugonnet et al. in 2009, have reported the efficient role of clavulanate against XDR-TB (113). A recent study by Diacon et al. reported the combination use of amoxicillin/clavulanic acid with carbapenems, which led to the reduction in *M.tb* burden (114). Despite the successful use of this beta-lactam antibiotic in TB, there is not much work on its effect on the immune system or on its immunomodulatory properties. Therefore, this area needs further research to successfully exploit this antibiotic as a repurposed drug against TB.

### Carbapenems

Carbapenems are beta-lactam antibiotics, which are unique in being impervious to being hydrolyzed by most beta-lactamases and being able to inhibit the PBP enzymes. The first beta-lactam antibiotic was isolated from *Streptomyces clavuligerus* followed by the development of clavulanic acid and thienamycin (115, 116). Thienamycin is considered the parent carbapenem, which has been modified to form all the subsequently discovered carbapenems. Of all beta-lactams synthesized, carbapenems have the broadest range of activity against both gram-positive and gram-negative bacteria making them “the drugs of last resort” (117, 118). To improve stability, thienamycin was chemically modified over time into other more stable derivatives such as imipenem (119). Later, more stable derivatives with a broader spectrum such as biapenem, meropenem, doripenem, and ertapenem were synthesized (120–125). Carbapenems target the PBP enzymes inhibiting peptidoglycan synthesis *via* crosslinking. Eventually, the bacterial wall weakens, leading to the death of the bacteria due to high osmotic pressure. Imipenem and panipenem act better against gram-positive bacteria whereas biapenem, meropenem and doripenem kill gram-negative bacteria efficiently (126–129). A combination study by Hugonnet et al., 2009 states that meropenem together with clavulanic acid kills MDR *M.tb* efficiently (113). Another study by Tiberi et al. reported that carbapenems when given intravenously are extremely helpful in treating XDR-TB strains (130). Veziris et al., in 2011 had previously shown that even though less efficient compared to INH, treatment with a combination of carbapenem together with clavulanate in *M.tb* infected mice increased the survival of the mice while reducing the bacterial load (131). As discussed earlier, a combination of amoxicillin/clavulanic acid and carbapenem has been studied in clinical trials and seem very promising for the treatment of DR-TB (114). In spite of being proposed as an adjunct therapy for DR-TB, there is almost no information on the effect of these drugs on the immune system and needs serious research.

### Sulfonamides and their Derivatives

The sulfonamides or sulfa drugs are wide spectrum bacteriostatic antibiotics, which work against most gram-positive and gram-negative bacteria. Sulfonamides and their derivatives were used from the 1930s up to the 1950s as a monotherapy (132) but were later discontinued due to their low efficacy compared to INH and streptomycin and high toxicity (133). It was believed that *M.tb* is resistant to trimethoprim-sulfamethoxazole (TMP-SMX). However, in 2009 a study in humans reported that the use of TMP-SMX on immuno-compromised patients provided better outcome and the drug worked on the *M.tb* strains isolated from the same infected patients (134). In another study conducted in 2014 in HIV-TB co-infected patients, who were being treated with TMP-SMX in order to protect them from *Pneumocystis jirovecii* infection, TMP-SMX proved to be quite effective in preventing TB (135). In another study conducted in Nigeria, in patients co-infected with HIV and MDR-TB, the time required for sputum conversion reduced significantly upon administration of TMP-SMX (136). Sulfadiazine, a sulfa drug used for the treatment of leprosy has been repurposed to treat DR-TB and proved to be more effective and safe than other sulfa drugs for the treatment of TB (137, 138). These drugs can be tested further to be included in TB treatment through more research using random human cohorts as subjects. Regarding the way they affect the immune system, this area needs more extensive study, as there are very limited information available which may establish their role as immunomodulators.

## Conclusions and Future Perspective

Drug repurposing is indisputably a smart strategy to develop a new treatment regime for TB within a short period of time and also to treat drug-resistant pathogens. Some of the repurposed drugs have shown great promise for future treatment of TB and have been extensively studied. However, we still need to repurpose as many drugs as we can through various approaches such as computational and experimental biology to explore the potential of already existing thousands of drugs in order to minimize the time for novel drug discovery as the incidence of resistance in the *M.tb* population is occurring at a very fast pace and we urgently need a new improved treatment regime. Such studies should be organized in the human cohorts. As the influence of the host-protective immune system continues to gain attention in the advancement of host-directed therapies so we should also aim to study how each of the repurposed drugs affects the balance of the host immune system and deals with infection and inflammation. This would enable better designing of combination therapies that would help achieve the goal of TB eradication program by shortening of the treatment regime and preventing drug resistance while being cost-effective for the populations.

## Author Contributions

SF wrote the manuscript. SF, AB, and VD edited the manuscript. AB and VD conceived of the hypothesis. All authors contributed to the article and approved the submitted version.

## Funding

We would like to acknowledge financial support from the Department of Science and Technology (DST) and the Science and Engineering Research Board (SERB), Department of Science and Technology (DST), Government of India. SF is the recipient of DBT-RA Fellowship and AB and VD are the recipient of DST-INSPIRE Faculty Fellowship (DST/INSPIRE/04/2014/002012 and DST/INSPIRE/04/2014/002069) VD is the recipient of Early Career Research Award from SERB: ND/DST/16/023. We also would like to thank the institutional financial support from the International Centre for Genetic Engineering and Biotechnology (ICGEB), New Delhi, India.

## Conflict of Interest

The authors declare that the research was conducted in the absence of any commercial or financial relationships that could be construed as a potential conflict of interest.
